# Association between serum free hemoglobin level and cerebral white matter hyperintensity volume in older adults

**DOI:** 10.1038/s41598-022-07325-x

**Published:** 2022-02-28

**Authors:** Dae Jong Oh, Jun Sung Kim, Subin Lee, Hee Won Yang, Jong Bin Bae, Ji Won Han, Ki Woong Kim

**Affiliations:** 1 Department of Psychiatry, Seoul National University College of Medicine, Seoul, South Korea; 2 Department of Psychiatry, SMG-SNU Boramae Medical Center, Seoul, South Korea; 3 Department of Neuropsychiatry, Seoul National University Bundang Hospital, Gyeonggido, South Korea; 4 Institute of Human Behavioral Medicine, Seoul National University Medical Research Center, Seoul, South Korea; 5 Department of Electrical and Computer Engineering, Seoul National University, Seoul, South Korea; 6 Department of Brain and Cognitive Science, Seoul National University College of Natural Sciences, Seoul, South Korea

**Keywords:** Cerebrovascular disorders, Neurodegeneration

## Abstract

The association between serum free hemoglobin (sfHb) level and white matter hyperintensity (WMH) volume is controversial. This study is to examine this association considering nonlinearity, sex dimorphism, and WMH type. We enrolled 704 older adults among the participants of the Korean Longitudinal Study on Cognitive Aging and Dementia and visitors to the Dementia Clinic of Seoul National University Bundang Hospital. We measured sfHb level in the venous blood and WMH volume (V_WMH_) using fluid-attenuated inversion recovery magnetic resonance images. The association between sfHb level and periventricular V_WMH_ was linear in men (linear regression; *β* **= ** − 0.18, *p* = 0.006) and U-shaped in women (restricted cubic spline; *F* = 6.82, *p* < 0.001). sfHb level was not associated with deep V_WMH_ in either sex. These findings were also observed in participants without anemia. To conclude, sfHb level is associated with periventricular V_WMH_ in older adults of both sexes. Maintaining an optimal sfHb level may contribute to the prevention of WMH.

## Introduction

White matter hyperintensity (WMH) is prevalent in older adults^1,2^; associated with various adverse outcomes such as stroke, dementia, cognitive decline, and mortality^3^; and attributed to hypoxia and arteriosclerosis^4,5^. The levels of hemoglobin, a key oxygen-transport protein, steadily decrease with advancing age^6^. Thus, a low hemoglobin level may contribute to the development of WMH in older adults by inducing hypoxia and arteriosclerosis^7,8^.

Although the World Health Organization (WHO) proposed ≥ 13.0 g/dL and ≥ 12.0 g/dL as normal serum free hemoglobin (sfHb) levels in men and women respectively^9^, the optimal sfHb levels for health-related outcomes were different from the normal sfHb levels proposed by the WHO in older adults. For example, the optimal sfHb levels for increasing survival were ≥ 13.7 g/dL in men and sfHb ≥ 12.6 g/dL in women^10^, and those for reducing hospitalization and mortality were 14.0–17.0 g/dL in men and 13.0–15.0 g/dL in women^11^.

Although sfHb may also influence the risk and severity of cerebral WMH, its effect was not consistent in previous studies. To our knowledge, optimal sfHb levels for preventing or reducing cerebral WMH have never been investigated. Three cross-sectional studies investigated the association of sfHb level with the volume of cerebral WMH (V_WMH_) but their results were not consistent along with the studies. sfHb level showed a U-shaped association with V_WMH_ in one study^12^ while did not show a linear or non-linear association with V_WMH_ in other two studies^13,14^. One cross-sectional study found no association between sfHb level and the severity of cerebral WMH measured by a semi-quantitative visual rating scale^15^. One prospective study found that sfHb level did not influence the change in the severity of cerebral WMH measured by a semi-quantitative visual rating scale over 5-year follow-up period in older adults^16^. These inconsistencies among the previous studies might be attributable to the differences in the characteristics of participants, such as the age and sfHb level. In addition, several methodological issues should be considered. First, none of the previous studies considered the type of cerebral WMH (periventricular WMH [PVWMH] versus deep WMH [DWMH]) in their analyses despite that PVWMH is more affected by cerebral hypoperfusion than DWMH^4,17^. Second, three previous studies did not consider the effect of sex on the association between sfHb level and the severity or volume of WMH^12–14^, despite that normal sfHb level and prevalence of anemia are different between men and women^6^. Third, two studies examined the linear relationship between sfHb level and V_WMH_ only^15,16^ despite that the relationship between them may be non-linear^12^. Fourth, two studies measured the severity of cerebral WMH using semi-quantitative visual ratings instead of V_WMH_^15,16^. Despite of its clinical usefulness, the visual ratings on the severity of cerebral WMH are less sensitive to subtle differences in V_WMH_ compared to the fully quantitative volumetric measures^18^.

This study investigated whether the association of sfHb with V_WMH_ are different by the type of cerebral WMH and the sex of the participants under following hypotheses: (a) lower sfHb level is associated with higher V_WMH_; (b) the association between sfHb level and V_WMH_ may differ between men and women; and (c) the association of sfHb level with the volume of PVWMH (V_PVWMH_) may be different from that with the volume of DWMH (V_DVWMH_).

## Methods

### Study design, setting, and participants

This cross-sectional study included 358 participants from the Korean Longitudinal Study on Cognitive Aging and Dementia (KLOSCAD) and 346 visitors to the Dementia Clinic at Seoul National University Bundang Hospital (SNUBH) from 2010 to 2020. The KLOSCAD is an ongoing prospective cohort study that randomly samples 6818 community-dwelling Korean older adults. The baseline assessment of the KLOSCAD was conducted from 2010 to 2012 and four consecutive 2-year follow-up assessments had been completed in 2013–2021^19^. The participants of KLOSCAD took one or more brain scan at the baseline or follow-up assessments. The first brain scan of each participant was collected for the current study when the participant took two or more brain scans. Data were collected from the follow-up assessments as well as the baseline assessment of the KLOSCAD because some participants took their first brain scan in the baseline assessment while others took their brain scan at one of follow-up assessments. All participants were aged 55 years or older. The participants with a history of hematological (e.g., myelodysplastic syndrome, multiple myeloma, and polycythemia vera) and other malignancies, gastrointestinal tract resection, traumatic brain injury, multiple sclerosis, brain tumor, intellectual disability, and any dementing illnesses were excluded. Compared to the participants from the KLOSCAD, those from the SNUBH Dementia Clinic were older and showed higher prevalence of mild cognitive impairment, systolic blood pressure, V_WMH_ and V_PVWMH_ but lower burden of comorbidities and better estimated glomerular filtration rate (Supplementary Table S1).

This study was approved by the Institutional Review Board of Seoul National University Bundang Hospital (B-0912-089-010). All participants were fully informed about the study protocol and provided written informed consent.

### Clinical assessments

Geriatric psychiatrists conducted face-to-face standardized diagnostic interviews, physical and neurologic examinations, and routine laboratory tests to all participants using the Korean version of the Consortium to Establish a Registry for Alzheimer’s Disease Assessment Packet Clinical Assessment Battery^20^. A consensus panel of geriatric psychiatrists determined the diagnoses of dementia according to the Diagnostic and Statistical Manual of Mental Disorders, 4th edition (DSM-IV) criteria. Trained research nurses assessed the prior and current history of medical illnesses and the burden of comorbidities using the Cumulative Illness Rating Scale (CIRS)^21^. The trained research nurses also measured the mean systolic and diastolic blood pressure and body mass index (BMI). The estimated glomerular filtration rate was calculated from the serum creatinine level, age, and sex^22^.

### Assessment of sfHb level and anemia

After overnight fasting, trained research nurses collected venous blood samples from the participants for routine laboratory tests, including those for complete blood cell counts, total and high-density lipoprotein cholesterol levels, chemistry panel, thyroid function, and serum folate and vitamin B12 levels and serological tests for syphilis screening. The sfHb concentration in the venous blood was measured by using an ADVIA 2120i system (Siemens, Germany) for the KLOSCAD samples and an XN-9000 Hematology Analyzer (Sysmex, Japan) for the Clinic samples. The anemia was defined as the sfHb level of < 13 g/dL in men and < 12 g/dL in women according to the World Health Organization criteria^9^.

### Magnetic resonance imaging scan acquisition and processing

We obtained brain magnetic resonance imaging (MRI) scans using a 3.0 Tesla scanner (GE SIGNA by GE Healthcare, Milwaukee, WI, USA). Images were acquired using a T1-weighted sequence (echo time = 3.68 ms, repetition time = 25.0 ms, sagittal slice thickness = 1.0 mm) and fluid-attenuated inversion recovery (FLAIR) (echo time = 160 ms, repetition time = 9900 ms, axial slice thickness = 3.0 mm). We measured the intracranial volume using Freesurfer software version 5.3.0.0 (http://surfer.nmr.mgh.harvard.edu).

To segment the WMH, first, we obtained FLAIR images of all participants and we applied a bias correction on the images to correct non-uniformities caused by the bias field owing to different tissue properties and physics of MRI, using the “Segment” tool from Statistical Parametric Mapping software version 8 (SPM8, Wellcome Trust Centre for Neuroimaging, London, UK). We then segmented WMH from bias-corrected FLAIR images by using a fully automated in-house code run on MATLAB 2014a (MATLAB and statistics Toolbox Release 2014a, MathWorks, Inc., Natick, MA) which has been previously shown to work on different protocols from different scanners without any parameter adjustment^23^. The algorithm started with the brain extraction from FLAIR images and computed the surrogate for the V_WMH_. The algorithm calculated the optimal threshold intensity for classifying WMH with intensities of normal distribution that corresponded to normal tissues and WMH by applying Bayesian decision rule. It then removed false positive WMH which was located at gray matter or non-cerebral region. We calculated the total V_WMH_. Meanwhile, we segmented lateral ventricle of every participant from T1-weighted MR images and measured intracranial volume using Freesurfer software version 5.3.0 (http://surfer.nmr.mgh.harvard.edu) which enables fully-automated surface-based cortical segmentation. The lateral ventricle was co-registered and the WMH images were segmented to estimate PVWMH and DWMH volume according to the distance rule^24^. We classified PVWMH as WMH within 13 mm from lateral ventricle, else was classified as DWMH.

### Statistical analysis

We compared the continuous variables and categorical variables between groups using Student’s t-tests and Pearson’s chi-square tests, respectively. We examined the association between sfHb level and cerebral V_WMH_ using linear regression and restricted cubic spline analyses. In both analyses, we adjusted for age, hypertension, diabetes, stroke, CIRS score, drinking status, smoking status, systolic blood pressure, BMI, total cholesterol level, high-density lipoprotein cholesterol level, glomerular filtration rate, and cohort as covariates. In these regression analyses, V_WMH_ was normalized to individuals’ intracranial volumes and log-transformed. As sensitivity analysis, we performed the same linear regression analysis and restricted cubic spline analysis in the following two subgroups of participants as in the total participants: (1) subgroup 1 without anemia or iron supplementation (N = 612) and (2) subgroup 2 without a history of stroke (N = 680). The knots for restricted cubic spline analyses were set at the 5th, 35th, 65th, and 95th percentiles^25^, and the sfHb levels at the knots were provided in Supplementary Table S2. We performed all statistical analyses using STATA 12.1 (StataCorp LP; College Station, Texas).

### Ethics approval

This study was performed in line with the principles of the Declaration of Helsinki. Approval was granted by the Institutional Review Board of Seoul National University Bundang Hospital (B-0912-089-010).

### Consent to participate

All participants were fully informed about the study protocol and provided written informed consent.

## Results

As summarized in Table 1, female participants had a lower sfHb level than male participants. However, the frequency of anemia was comparable between sexes. Female participants showed smaller intracranial volume and V_WMH_ than male participants. However, after adjusting for intracranial volume, the V_WMH_ was comparable between them (*p* = 0.201 for total WMH; *p* = 0.208 for PVWMH; *p* = 0.770 for DWMH).

**Table 1:** Characteristics of participants.

	Total (n = 704)	Male (n = 277)	Female (n = 427)	p*
Age, years, mean (SD)	73.8 (6.6)	73.7 (6.8)	73.8 (6.5)	0.923
Hypertension, n (%)	372 (52.8)	142 (51.3)	230 (53.9)	0.500
Diabetes mellitus, n (%)	138 (19.6)	61 (22.0)	77 (18.0)	0.193
Stroke, n (%)	24 (3.4)	17 (6.2)	7 (1.6)	0.001
CIRS score, mean (SD)	6.5 (3.1)	6.9 (3.1)	6.1 (3.0)	0.001
Current drinking, n (%)	47 (6.7)	46 (16.6)	1 (0.2)	< 0.001
Current smoking, n (%)	37 (5.3)	36 (13.0)	1 (0.2)	< 0.001
SBP, mmHg, mean (SD)	128.9 (14.3)	128.7 (14.0)	129.0 (14.5)	0.773
DBP, mmHg, mean (SD)	75.9 (9.1)	76.3 (8.7)	75.6 (9.3)	0.361
BMI, kg/m^2^, mean (SD)	23.9 (2.9)	24.0 (2.8)	23.9 (2.9)	0.467
Cholesterol, mg/dL, mean (SD)	183.7 (37.7)	176.3 (35.6)	188.5 (38.2)	< 0.001
HDL, mg/dL, mean (SD)	53.3 (13.5)	49.2 (12.1)	55.9 (13.6)	< 0.001
GFR, mL/min, mean (SD)	79.4 (18.5)	76.9 (16.7)	81.0 (19.5)	0.003
Hemoglobin, g/dL, mean (SD)	13.7 (1.4)	14.5 (1.4)	13.1 (1.1)	< 0.001
Anemia, n (%)	88 (12.5)	35 (12.6)	53 (12.4)	0.930
ICV, cc, mean (SD)	1508.8 (155.0)	1627.3 (137.4)	1431.8 (111.1)	< 0.001
V_WMH_, cc, mean (SD)	11.0 (15.7)	12.8 (18.1)	9.8 (13.7)	0.020
V_PVWMH_, cc, mean (SD)	10.2 (15.4)	11.9 (17.8)	9.1 (13.5)	0.025
V_DWMH_, cc, mean (SD)	0.8 (2.2)	0.9 (3.2)	0.8 (1.2)	0.358

*CIRS* cumulative illness rating scale, *SBP* systolic blood pressure, *DBP* diastolic blood pressure, *BMI* body mass index, *HDL* high-density lipoprotein cholesterol, *GFR* glomerular filtration rate, *ICV* intracranial volume, *V*_*WMH*_ volume of total white matter hyperintensity, V_PVWMH_ volume of periventricular white matter hyperintensity, *V*_*DWMH*_ volume of deep white matter hyperintensity.

*Sex-differences by Student t-tests for continuous variables and chi-square tests for categorical variables.

In male participants, the linear regression models demonstrated that sfHb level was negatively associated with the log-transformed V_WMH_ and V_PVWMH_. However, this association was not observed for the log-transformed V_DWMH_ (Table 2). Similar findings were observed for the restricted cubic spline models adjusted for the same covariates as those used in the linear regression models (Table 3).

**Table 2:** Linear association between serum free hemoglobin level and white matter hyperintensity volume.

	V_WMH_		V_PVWMH_		V_DWMH_	
	*β (SE)*	*P*	*β (SE)*	*p*	*β (SE)*	*p*
**All**						
Male						
Hb, unadjusted	** − 0.23 (0.06)**	** < 0.001**	** − 0.25 (0.06)**	** < 0.001**	− 0.04 (0.07)	0.534
Hb, adjusted*	** − 0.15 (0.06)**	**0.013**	** − 0.18 (0.06)**	**0.006**	− 0.07 (0.08)	0.350
Female						
Hb, unadjusted	− 0.09 (0.07)	0.160	− 0.11 (0.07)	0.127	0.03 (0.07)	0.692
Hb, adjusted*	− 0.06 (0.07)	0.355	− 0.08 (0.07)	0.259	0.06 (0.08)	0.483
**Subgroup 1**^**a**^						
Male						
Hb, unadjusted	** − 0.21 (0.09)**	**0.017**	** − 0.26 (0.10)**	**0.005**	0.004 (0.09)	0.964
Hb, adjusted*	− 0.15 (0.09)	0.113	** − 0.20 (0.10)**	**0.044**	− 0.11 (0.11)	0.332
Female						
Hb, unadjusted	− 0.07 (0.09)	0.420	− 0.09 (0.10)	0.361	0.15 (0.10)	0.147
Hb, adjusted*	− 0.12 (0.09)	0.194	− 0.14 (0.10)	0.136	0.12 (0.10)	0.269
**Subgroup 2**^**b**^						
Male						
Hb, unadjusted	** − 0.25 (0.06)**	** < 0.001**	** − 0.28 (0.06)**	** < 0.001**	− 0.04 (0.07)	0.540
Hb, adjusted*	** − 0.16 (0.07)**	**0.020**	** − 0.18 (0.07)**	**0.009**	− 0.06 (0.08)	0.473
Female						
Hb, unadjusted	− 0.09 (0.07)	0.171	− 0.10 (0.07)	0.137	0.03 (0.07)	0.683
Hb, adjusted*	− 0.07 (0.07)	0.324	− 0.08 (0.07)	0.234	0.05 (0.08)	0.509

*Hb* hemoglobin, *V*_*WMH*_ volume of total white matter hyperintensity, *V*_*PVWMH*_ volume of periventricular white matter hyperintensity, *V*_*DWMH*_ volume of deep white matter hyperintensity.

*Adjusted for age, hypertension, diabetes, stroke, cumulative illness rating sore, drinking status, smoking status, systolic blood pressure, body mass index, total cholesterol level, high-density lipoprotein cholesterol level, glomerular filtration rate, and cohort; the stroke was not adjusted as a covariate for the analysis of subgroup 2.

^a^Participants without anemia or iron supplementation.

^b^Participants without a history of stroke.

Significance values are given in bold.

**Table 3:** Restricted cubic spline model for association between serum free hemoglobin level and white matter hyperintensity volume.

	V_WMH_			V_PVWMH_			V_DWMH_		
	df	*F*	*p*	df	*F*	*P*	df	*F*	*p*
**All**									
Male									
Hb, unadjusted	**3**	**5.81**	** < 0.001**	**3**	**5.92**	** < 0.001**	3	0.57	0.635
Hb, adjusted*	**16**	**5.25**	** < 0.001**	**16**	**5.35**	** < 0.001**	16	0.51	0.938
Female									
Hb, unadjusted	3	1.87	0.135	3	1.84	0.139	3	1.61	0.186
Hb, adjusted*	**16**	**6.87**	** < 0.001**	**16**	**6.82**	** < 0.001**	16	1.39	0.146
**Subgroup 1**^**a**^									
Male									
Hb, unadjusted	**3**	**2.75**	**0.044**	**3**	**3.37**	**0.019**	3	0.53	0.659
Hb, adjusted*	**16**	**3.95**	** < 0.001**	**16**	**4.21**	** < 0.001**	16	0.52	0.936
Female									
Hb, unadjusted	3	2.10	0.100	3	2.05	0.107	3	1.16	0.325
Hb, adjusted*	**16**	**6.26**	** < 0.001**	**16**	**6.20**	** < 0.001**	16	1.40	0.139
**Subgroup 2**^**b**^									
Male									
Hb, unadjusted	**3**	**6.37**	** < 0.001**	**3**	**6.46**	** < 0.001**	3	0.70	0.551
Hb, adjusted*	**15**	**5.12**	** < 0.001**	**15**	**5.22**	** < 0.001**	15	0.75	0.734
Female									
Hb, unadjusted	3	1.92	0.125	3	1.89	0.130	3	1.77	0.153
Hb, adjusted*	**15**	**7.07**	** < 0.001**	**15**	**7.01**	** < 0.001**	15	1.36	0.166

*Hb* hemoglobin, *V*_*WMH*_ volume of total white matter hyperintensity, *V*_*PVWMH*_ volume of periventricular white matter hyperintensity, *V*_*DWMH*_ volume of deep white matter hyperintensity.

*Adjusted for age, hypertension, diabetes, stroke, cumulative illness rating sore, drinking status, smoking status, systolic blood pressure, body mass index, total cholesterol level, high-density lipoprotein cholesterol level, glomerular filtration rate, and cohort; the stroke was not adjusted as a covariate for the analysis of subgroup 2.

^a^Participants without anemia or iron supplementation.

^b^Participants without a history of stroke.

Significance values are given in bold.

**Figure 1:**
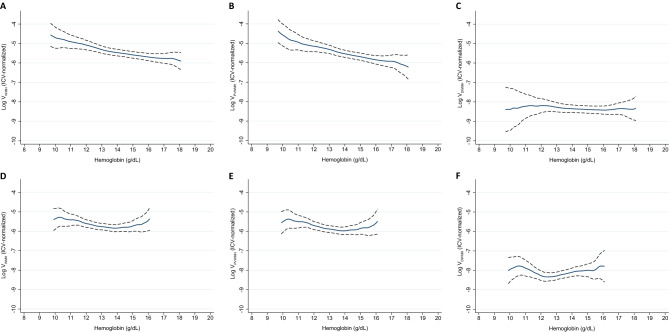
Serum free hemoglobin level and volumes of white matter hyperintensities. *ICV* intracranial volume* V*_*WMH*_ volume of total white matter hyperintensity, *V*_*PVWMH*_ volume of periventricular white matter hyperintensity, *V*_*DWMH*_ volume of deep white matter hyperintensity. Restricted cubic spline analyses for the association of serum free hemoglobin level with volumes of white matter hyperintensities. Volumes of total (**A**), periventricular (**B**), and deep (**C**) white matter hyperintensities and serum free hemoglobin level in male older adults, and volumes of total (**D**), periventricular (**E**), and deep (**F**) white matter hyperintensities and serum free hemoglobin level in female older adults. All the volumes were normalized to individuals’ intracranial volumes and log-transformed.

In female participants, the associations between sfHb level and the log-transformed V_WMH_, V_PVWMH_, and V_DWMH_ were not significant in the regression models. However, the associations between sfHb level and the log-transformed V_WMH_ and V_PVWMH_ were significant in the restricted cubic spline models adjusted for the same covariates as those used in the linear regression models, indicating that the association may be nonlinear (Table 3). As demonstrated in Fig. 1, which shows the fitted regression with 95% confidence intervals estimated from the restricted cubic spline models, the associations between sfHb level and the log-transformed V_WMH_ and V_PVWMH_ were linear in male participants and U-shaped in female participants.

These results did not change when we analyzed subgroups 1 and 2 separately (Tables 2, 3).

## Discussion

The results of this study in older adults showed that the association between sfHb level and V_WMH_ was sexually dimorphic and confined to PVWMH. The sfHb level showed a negative linear association with V_PVWMH_ in men and a U-shaped association in women. Furthermore, these associations remained significant when participants without anemia were analyzed separately.

To our knowledge, this is the first study to identify that the association between sfHb level and V_WMH_ differed by the type of WMH. Our findings demonstrated that a lower sfHb level was associated with increasing V_PVWMH_ only in both sexes. This result is in line with a previous study on stroke patients. In stroke patients, sfHb level was associated with V_PVWMH_, but not with V_DWMH_^26^. When the sfHb level decreases, cerebral blood flow increases to compensate for cerebral deoxygenation^7,27^. As the microvasculature in white matter is scarcer and its’ connection to the pial capillary plexus is longer, more indirect and tortuous than those in gray matter^28,29^, the compensatory increase of cerebral blood flow against deoxygenation associated with low sfHb level in white matter is far lower than that in cerebral gray matter^30^. Within cerebral white matter, periventricular white matter is more vulnerable to focal or systemic cerebral hypoperfusion than deep white matter because periventricular white matter has less collateral blood supplies for ventriculofugal vessels than deep white matter^31^. PVWMH was associated with decreased cortical blood flow but DWMH was not^17^. The current study suggests that dynamic autoregulation of cerebral blood flow against altered sfHb levels may be more decompensated in periventricular white matter than in deep white matter. The impaired dynamic autoregulation of cerebral blood flow is closely associated with the cerebrovascular risk factors known to increase dementia risk^32^, and that could partially explain why the PVWMH is more strongly associated with the risk of dementia than DWMH^5^. Maintaining optimal sfHb level may be beneficial to reduce the risk of PVWMH and cognitive decline associated with PVWMH in older adults.

This study also found the sexually dimorphic association between sfHb level and V_PVWMH_. Our finding is in line with a previous study reported a sexually dimorphic association between the risk of stroke and sfHb level. In that study, a U-shaped association between the risk of stroke and sfHb level was found in women only^33^. Regardless of sex, the increase in sfHb level may reduce cerebral blood flow by elevating blood viscosity and activating platelets^34^. In women, reduced blood estrogen levels by increasing age may weaken the ability to compensate for the reduced cerebral microcirculation associated with a high sfHb level, because estrogen is known to facilitate cerebral microvasculature dilatation and regulates the coagulation pathway^35,36^. In men, however, serum androgen which constricts brain microvasculature decreases with increasing age. Therefore, older men may have better ability to compensate for the reduced cerebral blood flow induced by high sfHb level than older women^36^.

The inconsistent results on the sfHb–WMH association in previous studies^12–16^ may be, at least in part, attributable to the dimorphisms in the sfHb–WMH association by sex and the type of WMH demonstrated in the current study. The sfHb–WMH association with increasing age might have been prominent specifically in this study because of the participants with higher age and lower sfHb level compared to the most previous studies^12–15^. In men with the sfHb level below the normal range and women with the sfHb level below or above the normal range, the risk of PVWMH and associated cognitive impairments needs to be evaluated and their abnormal sfHb should be quickly managed. In addition, optimal sfHb levels for men and women should be investigated in future research because the dimorphic sfHb–WMH association was observed in the older adults without anemia.

This study has several limitations. First, this is a cross-sectional design, which limits the interpretation of the causal relationship between sfHb level and V_WMH_. Second, this study analyzed data from two separate cohorts with different participants and measured sfHb levels using different automated systems. However, our analytic models included cohort as a covariate and still showed significant associations between hemoglobin with WMH. Third, the chronicity and the etiology of low or high sfHb levels were not identified. Finally, this study was limited to the Asian population. As the normal range of hemoglobin and the prevalence of red cell disorders are differentially distributed by ethnicities, our findings should be replicated in various populations.

## Summary

Our study provides evidence that sfHb level differentially affects V_WMH_ according to sex and lesion type. Older adults with abnormal sfHb levels may be at high risk of periventricular white matter lesions; thus, modifiable factors should be screened to prevent adverse outcomes related to WMH.

## Supplementary Information

Supplementary Tables.

## Data Availability

The data that support the findings of this study are available from the corresponding author upon reasonable request.
