# Supplementary material for: Association between serum free hemoglobin level and cerebral white matter hyperintensity volume in older adults
Source: Sci Rep. 2022 Feb 28;12:3296. doi: 10.1038/s41598-022-07325-x (PMC8885699; doi:10.1038/s41598-022-07325-x)
Supplement: Supplementary file 1 — Supplementary Tables. [file 41598_2022_7325_MOESM1_ESM.pdf]

Supplementary material with the article

Association between serum free hemoglobin level and cerebral white matter hyperintensity volume in older adults

Dae Jong Oh, Jun Sung Kim, Subin Lee, Hee Won Yang, Jong Bin Bae, Ji Won Han, Ki Woong Kim

Supplementary Table 1. The comparison of participants between two cohorts

	KLOSCAD (n = 358)	Dementia Clinic (n = 346)	p^*
Age, years, mean (SD)	73.2 (6.1)	74.3 (7.0)	0.028
Hypertension, n (%)	194 (54.2)	178 (51.4)	0.466
Diabetes mellitus, n (%)	73 (20.4)	65 (18.8)	0.592
Stroke, n (%)	16 (4.5)	8 (2.3)	0.116
CIRS score, mean (SD)	6.8 (3.1)	6.1 (2.9)	0.003
Current drinking, n (%)	29 (8.1)	18 (5.2)	0.126
Current smoking, n (%)	20 (5.6)	17 (4.9)	0.696
SBP, mmHg, mean (SD)	127.3 (14.0)	130.6 (14.4)	0.002
DBP, mmHg, mean (SD)	75.5 (9.1)	76.3 (9.1)	0.288
BMI, kg/m^2, mean (SD)	24.0 (2.8)	23.8 (3.0)	0.389
Cholesterol, mg/dL, mean (SD)	183.5 (36.9)	183.8 (38.6)	0.914
HDL, mg/dL, mean (SD)	52.5 (13.7)	54.0 (13.2)	0.138
GFR, mL/min, mean (SD)	76.5 (18.0)	82.5 (18.7)	<0.001
Hemoglobin, g/dL, mean (SD)	13.6 (1.4)	13.7 (1.4)	0.453
Anemia, n (%)	47 (13.1)	41 (11.8)	0.608
MCI, n (%)	84 (23.5)	243 (70.2)	<0.001
ICV, cc, mean (SD)	1515.4 (158.1)	1502.1 (151.7)	0.258
V_{WMH}, cc, mean (SD)	8.7 (12.2)	13.4 (18.2)	<0.001
V_{PVWMH}, cc, mean (SD)	7.7 (11.8)	12.7 (18.0)	<0.001
V_{DWMH}, cc, mean (SD)	0.9 (3.0)	0.7 (0.9)	0.193

KLOSCAD, The Korean Longitudinal Study of Aging; CIRS, cumulative illness rating scale; SBP, systolic blood pressure; DBP, diastolic blood pressure; BMI, body mass index; HDL, high-density lipoprotein cholesterol; GFR, glomerular filtration rate; MCI, mild cognitive impairment; ICV, intracranial volume, V_{WMH}, volume of total white matter hyperintensity; V_{PVWMH}, volume of periventricular white matter hyperintensity; V_{DWMH}, volume of deep white matter hyperintensity

**p*-values from Student t-tests for continuous variables and chi-square tests for categorical variables

Supplementary Table 2. The values of serum free hemoglobin levels for the knots of restricted cubic spline analyses

	Male	Female
All		
5th percentile (g/dL)	11.9	11.2
35th percentile (g/dL)	14.1	12.6
65th percentile (g/dL)	15.0	13.5
95th percentile (g/dL)	16.5	14.7
Subgroup 1[a]		
5th percentile (g/dL)	13.3	12.1
35th percentile (g/dL)	14.3	12.9
65th percentile (g/dL)	15.2	13.6
95th percentile (g/dL)	16.6	14.8
Subgroup 2[b]		
5th percentile (g/dL)	11.9	11.2
35th percentile (g/dL)	14.0	12.6
65th percentile (g/dL)	15.0	13.5
95th percentile (g/dL)	16.5	14.7

a. Participants without anemia or iron supplementation

b. Participants without a history of stroke
